# Localisation of cryptochrome 2 in the avian retina

**DOI:** 10.1007/s00359-021-01506-1

**Published:** 2021-10-22

**Authors:** Angelika Einwich, Pranav Kumar Seth, Rabea Bartölke, Petra Bolte, Regina Feederle, Karin Dedek, Henrik Mouritsen

**Affiliations:** 1 Institute for Biology and Environmental Sciences, Carl von Ossietzky University Oldenburg, Oldenburg, Germany; 2 Research Centre for Neurosensory Sciences, Carl von Ossietzky University Oldenburg, Oldenburg, Germany; 3 Helmholtz Zentrum München, German Research Center for Environmental Health, Institute for Diabetes and Obesity, Monoclonal Antibody Core Facility, Neuherberg, Germany

**Keywords:** Migratory orientation, Circadian clock, Magnetic compass, *Erithacus rubecula*, Chicken

## Abstract

Cryptochromes are photolyase-related blue-light receptors acting as core components of the mammalian circadian clock in the cell nuclei. One or more members of the cryptochrome protein family are also assumed to play a role in avian magnetoreception, but the primary sensory molecule in the retina of migratory birds that mediates light-dependent magnetic compass orientation has still not been identified. The mRNA of cryptochrome 2 (Cry2) has been reported to be located in the cell nuclei of the retina, but Cry2 localisation has not yet been demonstrated at the protein level. Here, we provide evidence that Cry2 protein is located in the photoreceptor inner segments, the outer nuclear layer, the inner nuclear layer and the ganglion cell layer in the retina of night-migratory European robins, homing pigeons and domestic chickens. At the subcellular level, we find Cry2 both in the cytoplasm and the nucleus of cells residing in these layers. This broad nucleic expression rather points to a role for avian Cry2 in the circadian clock and is consistent with a function as a transcription factor, analogous to mammalian Cry2, and speaks against an involvement in magnetoreception.

**Supplementary Information:**

The online version contains supplementary material available at 10.1007/s00359-021-01506-1.

## Introduction

Cryptochromes are blue-light receptors originally discovered in *Arabidopsis thaliana* (Ahmad and Cashmore 1993) but found throughout the biological kingdoms, from bacteria to insects and mammals (Sancar 2003; Lin and Todo 2005; Chaves et al. 2011). They are evolutionarily related to photolyases, which catalyse the blue-light-dependent repair of UV-damaged DNA (Sancar 1994, 2003). Like their photolyase ancestors, cryptochromes are flavoproteins: They often contain flavin adenine dinucleotide (FAD) as a covalently bound cofactor, and some cryptochromes also have a second light-harvesting chromophore (Cashmore et al. 1999; Sancar 2003). Cryptochromes, though, possess no DNA repair activity; instead, they are involved in the entrainment of circadian rhythms in vertebrates (Miyamoto and Sancar 1998; Cashmore et al. 1999; Sancar 2000, 2004). Circadian rhythms are established and maintained through transcription-translation feedback loops (TTFL), which consist of positive and negative regulatory elements. In mammals, positive elements of the TTFL are Clock, Npas2, Bmal1 and Cycle, whereas Period (Per) and Cryptochrome (Cry) proteins serve as negative elements (Takahashi 2017). Heterodimers of Clock and Bmal1 act as transcription factors to drive the rhythmic expression of Per and Cry genes, which in turn dimerise, translocate back into the nucleus and repress their own Clock/Bmal1-dependent transcription in the main feedback loop (Griffin et al. 1999; Kume et al. 1999; van der Horst et al. 1999). Apart from their core function in the circadian clock, vertebrate cryptochromes have also been proposed to play a role in avian magnetoreception (Ritz et al. 2000; Hore and Mouritsen 2016; Mouritsen 2018), which is thought to be based on a light-dependent (Wiltschko and Wiltschko 1993; Zapka et al. 2009; Wiltschko et al. 2010), quantum–mechanical mechanism involving radical pairs (Schulten et al. 1978; Ritz et al. 2000; Hore and Mouritsen 2016). It takes place in both eyes of birds (Hein et al. 2010, 2011; Engels et al. 2012), and magnetic signals are further processed in the bird’s thalamofugal visual pathway (Heyers et al. 2007; Zapka et al. 2009, 2010; Mouritsen et al. 2016). Since cryptochromes are the only proteins known in the vertebrate retina to be able to form radical pairs upon photoexcitation (Ritz et al. 2000; Maeda et al. 2008, 2012; Biskup et al. 2009; Hiscock et al. 2016), they are suggested to be the primary magnetoreceptors in the magnetic compass of migratory birds (Ritz et al. 2000; Hore and Mouritsen 2016). In mammals, two cryptochrome genes (*Cry1* and *Cry2*) exist and the resulting proteins, Cry1 and Cry2, are both involved in the circadian clock (Miyamoto and Sancar 1998). Birds, in contrast, possess three Cry genes, *Cry1*, *Cry2* and *Cry4*, coding for at least six different Cry proteins: Cry1a, Cry1b, Cry2a (formerly called Cry2; Yamamoto et al. 2001), Cry2b (Hochstoeger et al. 2020), Cry4a (formerly called Cry4) (Liedvogel and Mouritsen 2010) and Cry4b (Einwich et al. 2020). Both Cry2b and Cry4b have just recently been reported: Cry2b has been identified in the homing pigeon (Hochstoeger et al. 2020) and Cry4b has been found in the night-migratory songbird European robin (Einwich et al. 2020). Deducing from the amino acid identity, avian Cry1a and Cry2a/b are orthologues to mammalian Cry1 and Cry2, respectively. Cry1b, Cry2b and Cry4b seem to be bird-specific (Möller et al. 2004; Bolte et al. 2016; Nießner et al. 2016; Einwich et al. 2020; Hochstoeger et al. 2020), whereas Cry4(a) has been found in fish (Kobayashi et al. 2000), birds (Kubo et al. 2006, Liedvogel and Mouritsen 2010), amphibians (Takeuchi et al. 2014) and reptiles (Liu et al. 2015). During the past few years, the retinal location of the Cry proteins has been reported in several avian species. Cry1a was specifically found in the UV/V cone outer segments (Nießner et al. 2011; Bolte et al. 2021; Pinzon-Rodriguez and Muheim 2021), Cry1b is expressed in the photoreceptor inner segments, ganglion cells, and displaced ganglion cells (Bolte et al. 2016; Nießner et al 2016), and antibodies recognising both Cry4a and Cry4b proteins detected the Cry4 isoforms in the photoreceptor double cones and long-wavelength single cones in the European robin and the chicken (Günther et al. 2018). However, Hochstoeger et al. (2020) localised Cry4a in horizontal cells of the pigeon sretina. Currently, Cry4a is considered as the most probable candidate for being the light-dependent magnetoreceptor in the avian retina, both due to its location in the double cone outer segments (Hore and Mouritsen 2016; Worster et al. 2017; Günther et al. 2018, 2021), its ability to bind FAD (Öztürk et al. 2009; Wang et al. 2018; Zoltowski et al. 2019; Hochstoeger et al. 2020; Xu et al., 2021) and its magnetic-sensitive photochemistry demonstrated in vitro (Xu et al. 2021). Avian Cry2 proteins have been reported to possess a nuclear localisation signal (NLS), which presumably associates them to the nucleus (Möller et al. 2004; Mouritsen et al. 2004), but their location within the avian retina has not been demonstrated yet. On mRNA level, avian *Cry2a* has been identified in several tissues of both the chicken and the quail, including retina and pineal gland (Bailey et al. 2002; Fu et al. 2002). Specifically, *Cry2a* mRNA was found in the photoreceptor inner segments, the inner nuclear layer and the ganglion cell layer of the chicken retina (Bailey et al. 2002). A distribution of Cry2 proteins within or associated with the cell nuclei would rather speak against Cry2 as a magnetoreceptor, since a proper alignment that is essential for a directional magnetic field effect can only be achieved by tethering to the cytoskeleton or the membranes (Liedvogel and Mouritsen 2010; Hore and Mouritsen 2016), as outlined in Worster et al. (2017). Instead, both a nuclear location and a widely-distributed expression of the avian retinal Cry2 proteins would point to a role as a transcription factor of the circadian clock, analogous to mammalian Cry2 (Vitaterna et al. 1994; Thresher et al. 1998; Okamura et al. 1999; Griffin et al. 1999; Kume et al. 1999; van der Horst et al. 1999; Selby et al. 2000) since nuclear localisation appears to be a prerequisite for the transcriptional repressor activity of cryptochromes (Hirayama et al. 2003). Therefore, we analysed the subcellular location of the Cry2 proteins in the retina of migratory European robins, homing pigeons and domestic chickens using two different antibodies recognising both Cry2 isoforms. The aim of the present study is to investigate whether the Cry2 proteins, due to their location within the retina, are more likely to be involved in the circadian clock or if they might also play a different role, e.g. in magnetoreception.

## Material and methods

### Birds

Nine European robins (*Erithacus rubecula*) were wild-caught in the vicinity of the campus of the Carl von Ossietzky University Oldenburg using mist nets. One homing pigeon (*Columba livia domestica*) and three domestic chickens (*Gallus gallus domesticus*) were raised in the animal care facility of the University of Oldenburg. The birds were kept indoors under the natural light–dark cycle. Birds were sacrificed by decapitation to avoid the effects of injected substances on tissues of interest, or by an intramuscular injection of an overdose of Ketamin/Domitor (1 + 1 mix, 50–100 mg/kg bodyweight each). Eyes were prepared by cornea dissection followed by removal of the lens and vitreous bodies.

### Cloning

Retinae from freshly prepared eyecups were vortexed for 2 min in TRIzol Reagent (Life Technologies, Carlsbad, CA, USA), placed into liquid nitrogen and stored at − 80 °C. RNA samples were extracted according to the RNA preparation protocol for the TRIzol Reagent (Life Technologies), contaminating genomic DNA was digested with DNase I Amplification Grade (Invitrogen, Carlsbad, CA, USA). The cDNA synthesis was performed with the SmarterTM RACE cDNA Amplification Kit (Clontech Laboratories, Inc., Kalifornien, USA) according to the manufacturer’s protocol. An erCry2 specific primer (5′-AGCCCTGCCCCAAAGTGGAG-3′) was designed based on an already published erCry2 fragment (GenBank accession number AY7772689.1) to amplify the coding region of European robin Cry2a (erCry2a) by 5’RACE PCR. PCR products of the expected length were sub-cloned into the pGEM T easy vector (Promega, Madison, WI, USA) and subjected to sequencing (LGC, Berlin, Germany). By that, almost the entire erCry2a coding region was obtained, with only the first 39 nucleotides lacking (as assumed by alignment with the *Cry2* mRNA from other bird species available in BLAST databases). The sequence is available under the GenBank accession number MN656980.2. Cloning primers (sense: 5'-ATTCTCGAGATGTTTTGCCGCTCCGTGCA-3', with an artificial start codon included, and antisense: 5'-TCTAAGCTTGCACAAACTCTTCCCAGGGA-3') including restriction sites for *XhoI* and *HindIII* were used to amplify the coding regions with the RT-PCR kit GoTaq Long PCR Master Mix (Promega, Madison, WI, USA). The PCR products were cloned into the expression vector pTurboGFP-N (Evrogen, Moscow, Russia) according to the protocol for the Rapid DNA Dephos & Ligation Kit (Roche Diagnostics, Mannheim, Germany). The information about the missing N-terminal 39 nucleotides of the *erCry2a* ORF was taken from the published genome assembly of the European robin genome (GenBank accession number LR812108.1) (Feng et al. 2020). The full erCry2a sequence was then amplified with erCry2a-GFP in pTurbo as a template and a sense primer containing the missing 39 nucleotides (underlined) (5′ATATGCTAGCGGCCATTACGGCCATGGCGGCGGCGGCCGCTCTGGGCCCGGCGCCGGCCCTGTGCCGCTCCGTGCAC 3′), and the antisense primer (5′ GATATCTGCAGAATTCTCACAAACTCTTCCCAGGGATCTC 3′) using the CloneAmp HiFi PCR Premix (Takara Bio Inc., Shiga, Japan). The purified PCR product was then cloned into the *Sfi*I and *EcoR*I linearized mammalian expression vector pcDNA3.1^(+)^, modified to contain a Kozak sequence, an N-terminal deca-histidine tag and an *Sfi*I restricition site (gift from Prof. Karl-Wilhelm Koch, Carl von Ossietzky University Oldenburg), using the In-Fusion Snap Assembly Master Mix (Takara Bio Inc.).

### Antibodies

**Fig. 1:**
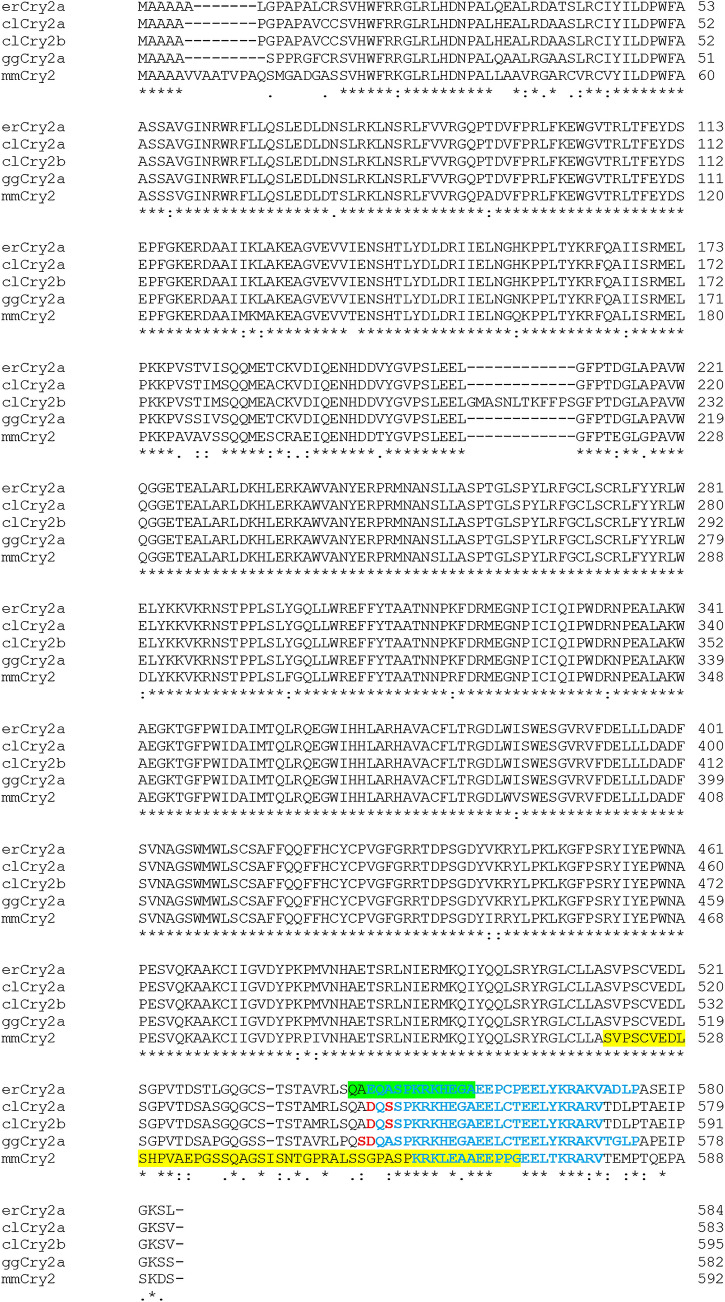
Alignment of the amino acid sequences of erCry2a, clCry2a, clCry2b, ggCry2a and mmCry2. The 14 amino acid long peptide from the European robin that the antibody erCry2-26E11 was raised against is shown in green (amino acid position 544 to 557 in erCry2), the 51 amino acid long peptide for mmCry2-A20 is shown in yellow (amino acid position 520 to 570 in mmCry2). All five Cry2 proteins possess a nuclear localisation signal (blue, bold). The amino acid sequences of clCry2a, clCry2b and ggCry2a have two mismatches to the peptide that the antibody erCry2-26E11 was raised against (marked in red). The multiple sequence alignment was performed using Clustal Omega (https://www.ebi.ac.uk/Tools/msa/clustalo/)

To localise the Cry2 proteins in the avian retina, two different anti-Cry2 antibodies were used to stain the retinae of six European robins, one homing pigeon and three domestic chickens. We produced a mouse monoclonal antibody, erCry2-26E11 (IgG2b/k), by immunisation of a 14 amino acid long peptide near the C-terminal end of erCry2 using hybridoma technology as described in Günther et al. (2018). This peptide sequence (12 out of 14 amino acids in total) is located within the NLS predicted by the NLS mapper (http://nls-mapper.iab.keio.ac.jp/cgi-bin/NLS_Mapper_form.cgi) (Fig. 1). A goat-derived polyclonal antibody, mmCry2-A20 from Santa Cruz Biotechnology, Inc. (Dallas, Texas, USA), had been raised against a 51 amino acid long peptide mapping near the C-terminus of the house mouse (*Mus musculus*) Cry2 (mmCry2, NCBI Reference Sequence: AF156987.1). The C terminal end is much less conserved between mouse and the birds than the core of the protein, but around 50% of the mmCry2 peptide sequence the mmCry2-A20 antibody was raised against is also conserved in the erCry2a protein (Fig. 1). The amino acid sequences of pigeon Cry2a (clCry2a, GenBank accession number: KX168609.1), clCry2b (GenBank accession number: KX168610.1) and chicken Cry2a (ggCry2a, NCBI Reference Sequence: NM_204244.1) are included in Fig. 1 to highlight mismatches to the two antibodies used in this study.

### Tissue preparation for immunohistochemistry

After preparation, eyecups were post-fixed in 4% PFA/PBS for 20–30 min and washed in PBS (pH 7.4). Tissue adaptation to cryoprotectant solution was performed overnight at 4 °C in 30% saccharose solution in 0.1 M PB (pH 7.4) and embedded in a cryoblock at − 20 °C. Vertical retinal sections of 20 µm were cut on a cryostat (CM 1950, Leica, Wetzlar, Germany), collected on gelatinized superfrost coverslips (Carl Roth, Karlsruhe, Germany), or Menzel SuperFrost Plus slides (Thermo Fisher Scientific, Waltham, MA, USA), and heat-fixed at 37 °C for one to three hours.

### Immunohistochemistry

After washing in 0.1 M phosphate buffer (PB), cryosections were blocked with 10% normal donkey serum (Sigma-Aldrich) in blocker mix (0.1 M PB containing 0.3% Triton X-100 and 2% normal donkey serum) for 30 to 60 min at room temperature. Incubation with primary antibodies diluted in blocker mix was performed at 4 °C overnight. The affinity-purified polyclonal antibody mmCry2-A20 was used in a dilution of 1:500. Monoclonal antibody erCry2-26E11 was used as unpurified hybridoma supernatant diluted 1:2 for the European robin and the homing pigeon and 1:3 for the domestic chicken. Due to the use of monoclonal hybridoma supernatant instead of affinity purified antibody, a low dilution was applied. For the pre-adsorption control, the antibody erCry2-26E11 was incubated for four hours at room temperature with 500 µg/ml Cry2 or Cry4 peptide (as a control) and then applied to the slices. After washing with 0.1 M PB, the slices were incubated for one hour with the secondary antibodies (Invitrogen, Carlsbad, CA, USA; for the European robin and the homing pigeon: Alexa 488 donkey anti-mouse and Alexa 568 donkey anti-goat, for the domestic chicken: Alexa 568 donkey anti-mouse and Alexa 647 donkey anti-goat), diluted 1:500 in blocker mix. After washing, slices were covered with Aqua-Poly/Mount (Polysciences, Inc., Warrington, PA, USA) and a cover slip. No measures to reduce potential autofluorescence were necessary as it was found to be weak, apart from the well-known fluorescence of oil droplets (e.g., Fig. 4l).

### Cell culture and protein expression

N2A and HEK293 cells were maintained in DMEM with 10% fetal calf serum and 1% antibiotics/antimycotics (Sigma-Aldrich). For immunocytochemistry, cells were plated in 24-well plates on glass coverslips that had been treated with poly L-Lysine for 30 min. For the immunoblot, HEK293 cells were seeded in 6-well plates (Roth). ErCry1a (Bolte et al. 2016), erCry2a, lacking the first 39 nucleotides and erCry4a (Günther et al. 2018) were expressed as fusion proteins with GFP from the respective generated pTurbo-GFP-N vectors using 2 µl Lipofectamine 2000 reagent (Invitrogen) per µg DNA in OptiMEM medium (Life Technologies, Carlsbad, CA, USA) following the manufacturer’s protocol.

### Immunocytochemistry of transfected cells

Forty hours after transfection, the cells were washed in phosphate buffered-saline (PBS) followed by a fixation with ice-cold methanol (7 min). After washing, the primary antibodies were incubated at 4 °C overnight (erCry2-26E11 diluted 1:3, mmCry2-A20 diluted 1:500 in PBS). After washing, the cells were incubated for two hours with appropriate secondary antibodies (Alexa 555 goat anti-mouse and Alexa 568 donkey anti-goat, diluted 1:500 in PBS) and mounted with Vectashield Antifade Mounting Medium with DAPI (Vector Laboratories Inc.).

### Immunoblot

Forty-eight hours after the transfection, the HEK293 cells were harvested from the wells and mechanically homogenised in buffer (50 mM Tris/HCl, pH 7.4, 2 mM EGTA, pH 7.4, 2 mM EDTA, pH 7.4, 0.1 mM sodium orthovanadate, 1 mM DTT, 2 μg/ml leupeptin, 5 μg/ml aprotinin, 2 mM PMSF in DMSO, Roche cOmplete™ Protease Inhibitor Cocktail). Some of it was stored at − 20 °C until usage whereas the rest was centrifuged (15 min, 8,000 rpm, 10 °C) until it had separated into a pellet and a supernatant. Total protein concentration of the total homogenate and the supernatant was measured by the Neuhoff assay (Neuhoff et al. 1979). The pellet was suspended in 8 M urea until the solution had become viscous. Both the supernatant and the pellet solution were used as samples.

For the immunoblot, the samples were mixed with SDS buffer (10% glycerol, 2% SDS, 1% beta-mercaptoethanol, 50 mM Tris–HCl pH 6.8; 12.5 mM EDTA pH 8.0, 0.02% bromophenol blue) and heated for four minutes at 95 °C. Ten to 15 µg of the samples were loaded on a standard SDS-PAGE (7.5% acrylamide, with a 4% acrylamide stacking gel). Gel electrophoresis was performed in an Eco Mini Buffer Tank (Biorad) using a 150 to 160 V separation current. Protein transfer from gel to membrane was performed in a wet blot system (350 mA for 60 min). After blocking of the nitrocellulose membrane (Whatman, Sigma-Aldrich) for one hour in 5% milk powder (Roth) in TBS-Tween, the membranes were incubated with the erCry2-26E11 primary antibody hybridoma supernatant (1:5 dilution) or a polyclonal GFP antibody (rabbit; Evrogen, Cat No AB513, Lot No 51301121268, 1:1000 dilution) diluted in 5% milk powder (Roth) in TBS-Tween overnight at 4 °C. After incubation with HRP-conjugated secondary antibodies (HRP anti-mouse or HRP anti-rabbit, dilutions 1:10,000), a chemiluminescence procedure was performed with Super Signal West Pico chemiluminescent substrates (Thermo Fisher Scientific) and the Image Lab Software version 5.2.1 (BioRad).

### Confocal microscopy and image processing

Confocal micrographs of fluorescent retinae and transfected cells were analysed with a confocal laser scanning microscope (TCS SP8, Leica) using the 488, 555 and 647 nm laser lines. Scanning was performed with the oil immersion 40 × HPX PL APO (NA 1.25 or 1.3) objective. Pixel size was adjusted for each experiment. All images of the same experiment were taken with the same microscope settings. Confocal stacks were subtracted in background, median-filtered (stainings with DAPI), and normalised in contrast in Fiji (https:\\fiji.sc) (Schindelin et al. 2012). Unless stated otherwise, all figures show maximum projections of confocal stacks, adjusted for contrast and brightness for presentation purposes.

## Results

Due to the recent report of a second isoform of avian Cry2 by Hochstoeger et al. (2020), we use the following terminology throughout this paper: ‘Cry2’ is used when the two isoforms cannot be distinguished, as it is the case for the immunohistological stainings. For the control experiments, however, we only worked with the erCry2a isoform, which we indicate as such.

**Fig. 2:**
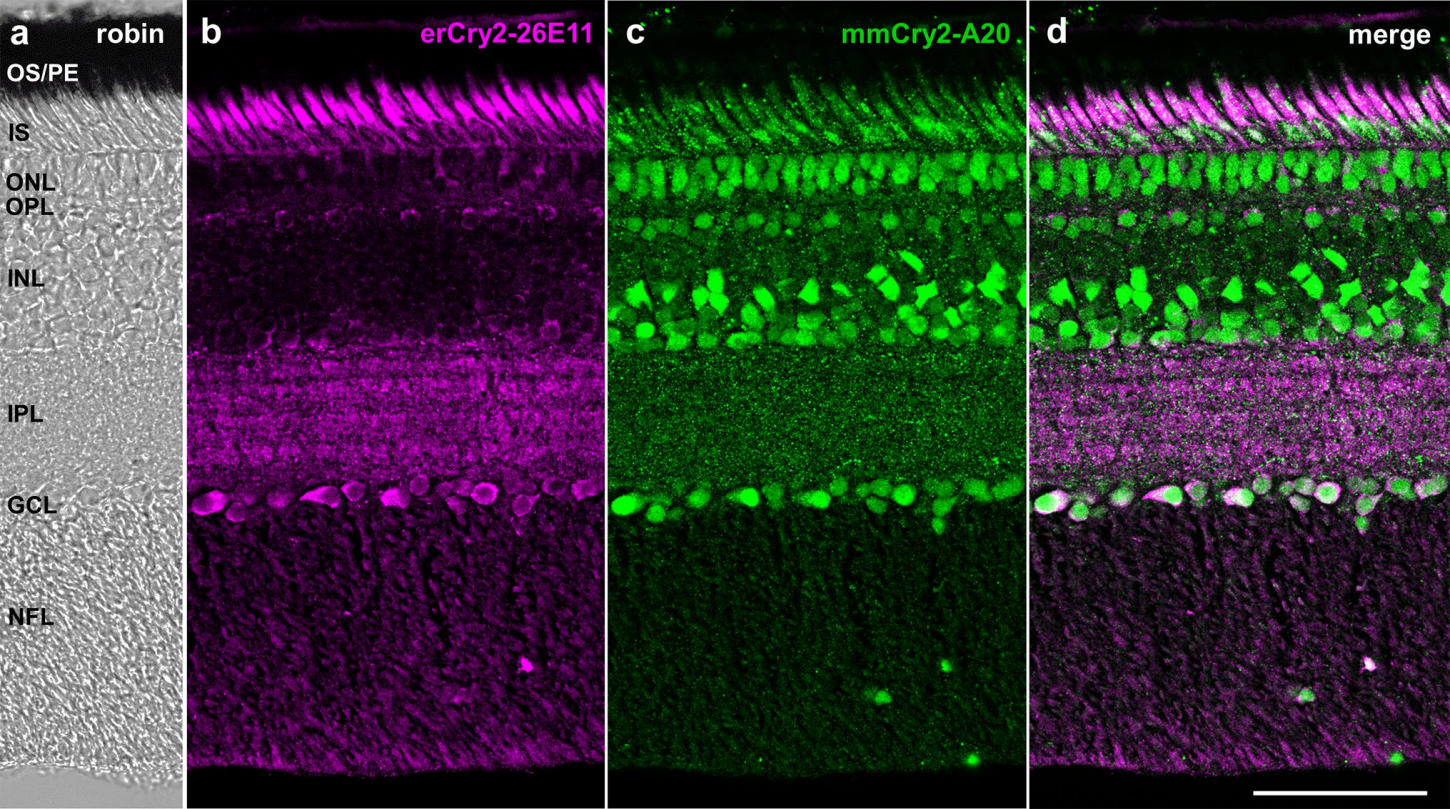
Cry2 localisation in the retina of the European robin. In the European robin retina (**a**), Cry2 was detected in the photoreceptor inner segments, outer nuclear layer, inner nuclear layer and ganglion cell layer (**b**–**d**). Whereas the erCry2-26E11 antibody labelled the ellipsoid of the inner segments as well as the cytoplasm of cells in the outer, inner and ganglion cell layers (**b**), mmCry2-A20 recognised more prominently the myoid of the photoreceptor inner segments as well as the nuclei of outer, inner and ganglion cell layers (**c**). Image **a** is a transmission image, images **b**–**d** are maximum projections of confocal stacks (z-size 1.27 µm, 7 sections). Scale bar: 50 µm. *OS* photoreceptor outer segments, *PE* retinal pigment epithelium, *IS* photoreceptor inner segments, *ONL* outer nuclear layer, *OPL* outer plexiform layer, *INL* inner nuclear layer, *IPL* inner plexiform layer, *GCL* ganglion cell layer, *NFL* nerve fibre layer

**Fig. 3:**
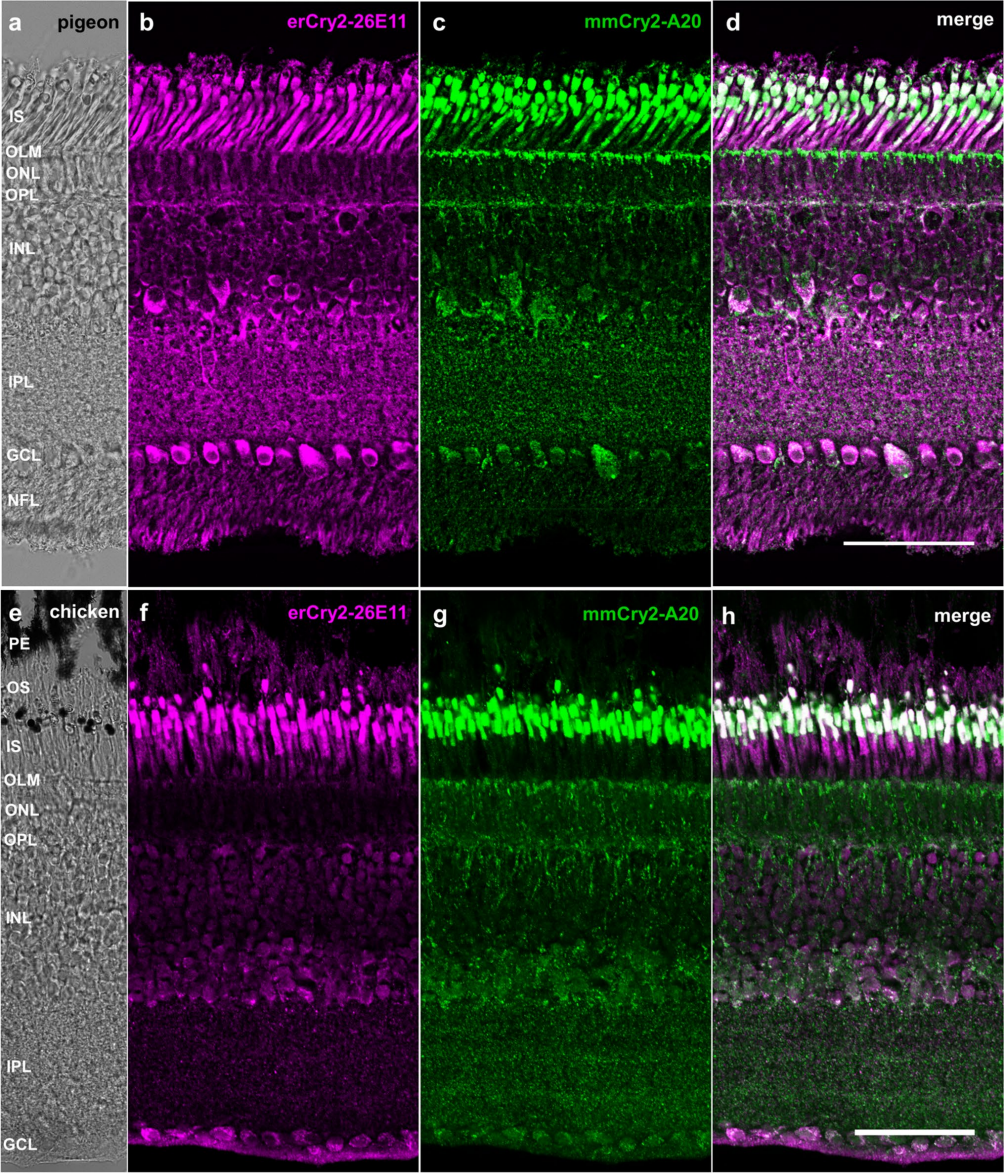
Cry2 location was confirmed in two additional bird species. The antibodies erCry2-26E11 and mmCry2-A20 stained the photoreceptor inner segments as well as the cytoplasm of cells in the inner nuclear layer and ganglion cell layer in the day-flying pigeon (**a**–**d**) and the domestic chicken (**e**–**h**). mmCry2-A20 was also labelling the outer limiting membrane in the pigeon (**c**) and the chicken (**g**). In contrast to the immunosignals observed in the European robin retina, mmCry2-A20 did not label the nuclei but the cytoplasm in pigeon and chicken. Images **a** and **e** are bright-field images, images **b**–**d** and **f**–**g** are maximum projections of confocal stacks (**b**, **c**: z-size 4 µm, 20 sections; **f**–**h**: z-size 2 µm, 10 sections). Scale bars: **d**, **h** 50 µm. *PE* retinal pigment epithelium, *OS* photoreceptor outer segments, *IS* photoreceptor inner segments, *OLM* outer limiting membrane, *ONL* outer nuclear layer, *OPL* outer plexiform layer, *INL* inner nuclear layer, *IPL* inner plexiform layer, *GCL* ganglion cell layer, *NFL* nerve fibre layer

In the retina of European robin, the two anti-Cry2 antibodies applied in this study, our custom-made mouse monoclonal antibody erCry2-26E11 and the commercially available polyclonal goat antibody mmCry2-A20 (Fig. 1), stained the photoreceptor inner segments as well as the outer nuclear layer, inner nuclear layer and ganglion cell layer (Fig. 2). Interestingly, the two Cry2 antibodies labelled the same cell types, but different regions of them: erCry2-26E11 seemed to stain predominantly the cytoplasm of the outer nuclear layer, inner nuclear layer and ganglion cell layer, whereas mmCry2-A20 staining was restricted to the nuclear part of those cells. The antibodies also showed a divergent signal in the photoreceptor inner segments: erCry2-26E11 signal was more prominent at the apex of the inner segment (the ellipsoid), which is rich in mitochondria in all vertebrate photoreceptors (Cohen 1972; Rodieck 1973; Stone et al. 2008), whereas mmCry2-A20 stained the lower region, called the myoid, where the paraboloid, endoplasmic reticulum, Golgi complex and ribosomes are located (Cohen 1972). A similarly differentiated staining of the Cry2 antibodies could also be observed in the pigeon retina (Fig. 3a–d). Here, erCry2-26E11 labelled the inner segments and, like in the European robin, the cytoplasm of cells in the inner nuclear layer and ganglion cell layer (Fig. 3b). In contrast, mmCry2-A20 recognised the ellipsoid of the inner segments and additionally the outer limiting membrane. Moreover, mmCry2-A20 seemed to stain both the cytoplasm and nuclei in the inner nuclear layer and ganglion cell layer (Fig. 3c). In the chicken, both antibodies recognised the inner segments and the cytoplasm of cells in the inner nuclear layer and the ganglion cell layer (Fig. 3e–h). The staining in the outer nuclear layer was only very faint with erCry2-26E11, whereas mmCry2-A20 seemed to recognise the outer limiting membrane (Fig. 3g). The staining pattern of the photoreceptor inner segments was again slightly diverging in chicken: erCry2-26E11 labelled the entire inner segments (with a stronger signal in the upper, ellipsoid part; Fig. 3f), whereas mmCry2-A20 exclusively and very strongly marked the ellipsoid part (Fig. 3g).

**Fig. 4:**
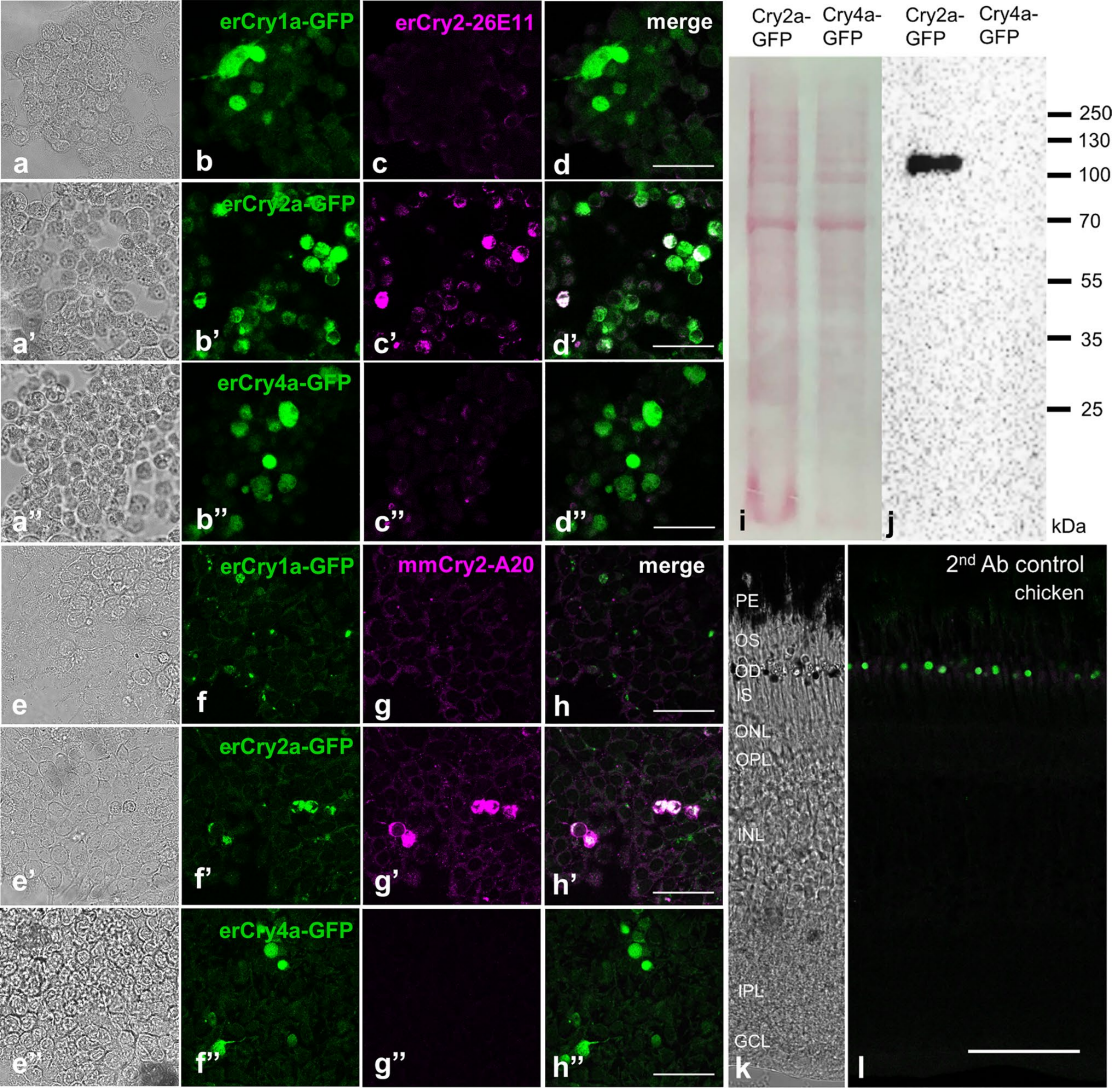
Controls for the antibodies. Staining of N2A cells (**a**-**a**’’) and HEK293 cells (**e**-**e**’’) expressing erCry1a-GFP (**b**, **f**), erCry2a-GFP (**b**’, **f**’) or erCry4a-GFP (**b**’’, **f**’’) protein with either our custom-made erCry2-26E11 antibody (**c**–**c**’’) or the commercially available mmCry2-A20 (**g**-**g**’’), respectively, indicates that both antibodies detected erCry2a-GFP (**d**’, **h**’) but not the other erCry-GFP proteins (**d**, **d**’’, **h**, **h**’’). Since all images are scanned and processed with the same parameters, some of the antibody signals are not visible in the resulting figure image. Immunoblot on recombinantly expressed erCry2a- and erCry4a-GFP fusion proteins (Ponceau staining, **i** incubated with the erCry2-26E11 antibody showed one single band only in the erCry2a-GFP lane (**j**). When omitting the primary antibodies in immunohistochemistry, no immunosignal was detected (**k**, **l**). Images **a**-**a**’’, **e**-**e**’’ and **k** are bright-field images; images **b**-**d**’’, **f**–**h**’’ and **l** are single confocal scans. Scale bars: 50 µm. *PE* retinal pigment epithelium, *OS* photoreceptor outer segments, *OD* oil droplets, *IS* photoreceptor inner segments, *ONL* outer nuclear layer, *OPL* outer plexiform layer, *INL* inner nuclear layer, *IPL* inner plexiform layer, *GCL* ganglion cell layer. For the immunoblot, the total homogenate of the protein samples was used

We also included co-stainings of the monoclonal antibody erCry2-26E11 with the nuclear marker DAPI to analyse the subcellular localisation of Cry2 in the retina of the European robin (Fig. S1), the domestic pigeon (Fig. S2) and the domestic chicken (Fig. S3). In all three bird species, labelling of Cry2 and DAPI revealed that Cry2 seems to be more prominently located in the cytoplasm, but was also detected in the nuclei of the ganglion cell layer (Fig. S1-S3e-g), albeit very weakly in the European robin (Fig. S1e-g). The specificity of the Cry2 antibodies was confirmed in several control experiments. After pre-adsorbing the Cry2-26E11 antibody with the respective erCry2 peptide, we obtained no Cry2 signal (Fig. S4c). In contrast, we still obtained a signal when pre-adsorbing the antibody with an erCry4 peptide (Fig. S4d), demonstrating that the Cry2 antibody specifically binds to its target epitope. Immunocytochemical stainings on N2A cells (Fig. 4a-a’’) and HEK293 cells (Fig. 4e-e’’) recombinantly over-expressing either erCry1a-, erCry2a- or erCry4a-GFP fusion proteins (Fig. 4b-b’’, f-f’’) showed that both antibodies, erCry2-26E11 (Fig. 4c-c’’) and mmCry2-A20 (Fig. 4g-g’’), detect the erCry2a-GFP protein (Fig. 4d’, 4 h’), but not the other erCry-GFP proteins (Fig. 4d, d’’, h, h’’). In immunoblot analysis (Fig. 4i, j), erCry2-26E11 detected a single protein in cell lysates of recombinantly expressed erCry2a-GFP but not erCry4a-GFP fusion protein (Fig. 4j). The band was running around 25 kDa higher than the expected size of 934 kDa, which might be due to posttranslational modifications, such as phosphorylation, and/or protein oligomerisation that could alter the molecular weight of the erCry2-GFP protein. By staining with an anti-GFP antibody, we confirmed that the larger than expected band is, in fact, the Cry2a-GFP fusion protein (Fig. S5). We also confirmed that the secondary antibodies did not produce any artefacts in immunohistochemical stainings of the avian retinae (secondary antibody control; here shown for the chicken retina, Fig. 4k, l). With the commercial antibody mmCry2-A20, however, we failed to obtain Cry2 signals on the immunoblot. Unfortunately, this antibody is no longer produced by Santa Cruz Biotechnology, so that we could not determine whether the antibody does not work in immunoblot experiments in general or whether this only affected our antibody lot. We were, therefore, also unable to perform pre-adsorption control experiments for the mmCry2-A20 antibody. Yet, the mmCry2-A20 antibody showed Cry2-specific labelling in immunocytochemical controls (Fig. 4g-g’’) and therefore, the staining in retinal tissue was regarded as specific. It is often the case that antibodies work in immunohistochemistry but not in immunoblot. One potential reason might be that the mmCry2-A20 recognized complex 3D structures that are preserved in fixed tissue but are denatured during sample preparation for immunoblotting.

## Discussion

It is generally accepted that members of the cryptochrome family are involved in the circadian clock (Miyamoto and Sancar 1998; Chaves et al. 2011), but they are also candidates for light-dependent magnetoreception (Ritz et al. 2000; Mouritsen et al. 2004; Hore and Mouritsen 2016; Xu et al. 2021). In this study, we aimed to find out where the Cry2 proteins are expressed in the avian retina since their subcellular location might provide evidence for their probable molecular function. Hence, we included three different bird species with differing magnetosensitive behaviour: night-migratory European robins, day-flying homing pigeons, and non-migratory domestic chickens. Furthermore, as we do not have the possibility to work with knock-out (migratory) birds, it is important to use different antibodies, ideally recognising different peptide epitopes of the same protein to confirm an antibody staining (Uhlen et al. 2016; Günther et al. 2018; Bolte et al. 2021). Here, we applied two different antibodies that were both raised against peptides near the C-terminal end of the European robin or mouse Cry2 proteins, respectively. Both antibodies should recognise both Cry2 protein isoforms, Cry2a and the recently reported bird-specific Cry2b protein (Hochstoeger et al. 2020). The mouse-specific polyclonal mmCry2-A20 antibody was raised against a considerably longer peptide (51 amino acids) than the European robin-specific antibody erCry2-26E11 (14 amino acids), but the sequences partially overlap. With both antibodies, we found Cry2 proteins ubiquitously expressed in the photoreceptor inner segments as well as in most, if not all cells of the nucleic cell layers (outer nuclear, inner nuclear and ganglion cell layers) in the retinae of all three bird species. This pattern is very similar to the pattern seen in mouse, where the expression of several clock proteins (Clock, Bmal1, Npas1, Per1, Per2, and Cry2) was observed prominently in cells of the inner nuclear layer and the ganglion cell layer, but also in a few cells in the outer nuclear layer (Liu et al. 2012). However, how Cry2 protein expression compares to the expression of other clock proteins in the avian retina, we cannot tell because to our knowledge, the expression of the clock genes has only been studied on the mRNA level, but not on the protein level so far (Yoshimura et al. 2000; Bian et al. 2019; Renthlei and Trivedi 2019). In our stainings, though, we detected slight differences between both antibodies and bird species: erCry2-26E11 showed stronger Cry2 labelling in the cytoplasm than in the cell nuclei in all three species (S1-Fig. S3e-g), whereas mmCry2-A20 recognised Cry2 in the cytoplasm of the pigeon and the chicken retina but in the cell nuclei of the European robin retina. A distribution of Cry2 in both cellular compartments is not per se surprising as protein synthesis takes place in the cytoplasm. In the circadian clock of mammals, freshly synthesised Cry2 (as well as Cry1) proteins heterodimerise with Per proteins in the cytoplasm and translocate to the nucleus, where they repress their own transcription (Kume et al. 1999). In accordance with that, human Cry2 protein has been found in both the cytoplasm and the nuclei of the retinal ganglion cell layer (Thompson et al. 2003). The exclusively cytoplasmic location we observed with the mmCry2 antibody in the pigeon and the chicken retina might be explained by masking effects. For instance, the antigenic site of the Cry2 protein may be masked by Per when located in the nucleus as a complex. Mammalian Cry1 and Cry2, however, have been shown to interact with Per1 and Per2 proteins at their photolyase homology region (Miyazaki et al. 2001; Ozber et al. 2010; Tomita et al. 2010), so that an interaction with Per proteins should probably not hide the antigenic site of avian Cry2 either. The two Cry2 antibodies used in this study also labelled the photoreceptor inner segments in all three birds species examined. Since the inner segment serves as the major housekeeping compartment of the photoreceptor cell (Pearring et al. 2013), where protein biosynthesis takes place, the Cry2 antibodies might recognise the freshly synthesised Cry2 proteins there. Since our antibodies are directed against both isoforms of avian Cry2, however, we cannot make any statement about a possible diverging localisation of Cry2a and Cry2b proteins in the bird retina. We observed species- and antibody-specific differences, though: In the European robin and the chicken, erCry2-26E11 showed a stronger signal in the ellipsoid of the inner segments, which is rich in mitochondria in all vertebrate photoreceptors (Cohen 1972; Rodieck 1973; Stone et al. 2008). In contrast, more or less the entire inner segment was stained by erCry2-26E11 in the pigeon retina. The sequence that this erCry2-specific antibody was raised against is well conserved in pigeon and chicken Cry2 (two amino acid mismatches out of 14; see Fig. 1). We also confirmed the specificity of both antibodies in several control experiments, which makes us confident that these stainings are reliable. The antibody mmCry2-A20 also shows species-specific differences in the photoreceptor inner segment stainings, but these differed from the pattern obtained with the erCry2-26E11 antibody: In European robins, mmCry2-A20 labelled the myoid, where paraboloid, endoplasmic reticulum, Golgi complex and ribosomes are located (Cohen 1972), whereas in the pigeon and the chickens, the signal was restricted to the ellipsoid region. The species-specific difference of Cry2 location in European robin *versus* pigeon and chicken retina could mean that the *Cry2* gene functions have diverged slightly between the (night-migratory) European robin and the (non-migratory) pigeon and chicken.

Their distribution within the cell nuclei of nearly every cell type of the retina could be a hint that the Cry2 proteins are involved in the circadian clock and would also speak against a role of Cry2 in avian magnetic compass orientation. Both proper fixation and alignment of the primary sensory protein(s) (which is hardly possible in the cell nuclei) are pre-requisites for light-dependent magnetoreception since this is needed for accurately detecting directional information (Soloy’yov et al. 2010; Hore and Mouritsen 2016; Hiscock et al. 2016; Worster et al. 2017). Furthermore, we would not expect a magnetoreceptive protein to be located within every retinal cell type. Sensory proteins are usually located in specialised sensory cells connected to the brain via a specific neuronal sensory pathway. However, since most proteins possess multiple functions (Jeffery 2020), we cannot entirely exclude that Cry2 acts as a magnetoreceptor in one cell type while it plays another role in other cell types. Moreover, since we could not distinguish between the two Cry2 isoforms with our antibodies, we cannot exclude that one of the isoforms is expressed exclusively in one specific cell type. Yet, currently available findings suggest that Cry2 is unable to bind FAD (Öztürk et al. 2009; Kutta et al. 2017), but FAD is essential for generating radical pairs and for any possible magnetic sensitivity of a Cry protein (Henbest et al. 2008; Maeda et al. 2012; Evans et al. 2013; Zollitsch et al. 2018; Xu et al. 2021). Together with recent independent reports that avian *Cry2a/b* mRNA displays a rhythmic circadian expression pattern characteristic of a circadian clock protein (Günther et al. 2018; Pinzon-Rodriguez et al. 2018; Hochstoeger et al. 2020), we conclude that the Cry2 proteins most likely act as core components of the circadian clock, not only in mammals but also in birds.

## Supplementary Information

Below is the link to the electronic supplementary material.

Supplementary file1 (PNG 152 kb)

Supplementary file2 (PNG 1281 kb)

Supplementary file3 (PNG 1571 kb)

Supplementary file4 (PNG 575 kb)

Supplementary file5 (DOC 19553 kb)

## Data Availability

The *erCry2a* mRNA, complete cds, is available under the GenBank accession number MN656980.2.

## Abbreviations

cl: *Columba livia domestica* (Homing pigeon)
Cry: Cryptochrome
er: *Erithacus rubecula* (European robin)
FAD: Flavin adenine dinucleotide
gg: *Gallus gallus domesticus* (Domestic chicken)
mm: *Mus musculus* (House mouse)
NLS: Nuclear localisation signal
PB: Phosphate buffer
PBS: Phosphate-buffered saline
Per: Period
TTFL: Transcription–translation feedback loop
