# Supplementary figures and images for: Localisation of cryptochrome 2 in the avian retina
Source: J Comp Physiol A Neuroethol Sens Neural Behav Physiol. 2021 Oct 22;208(1):69–81. doi: 10.1007/s00359-021-01506-1 (PMC8918457; doi:10.1007/s00359-021-01506-1)

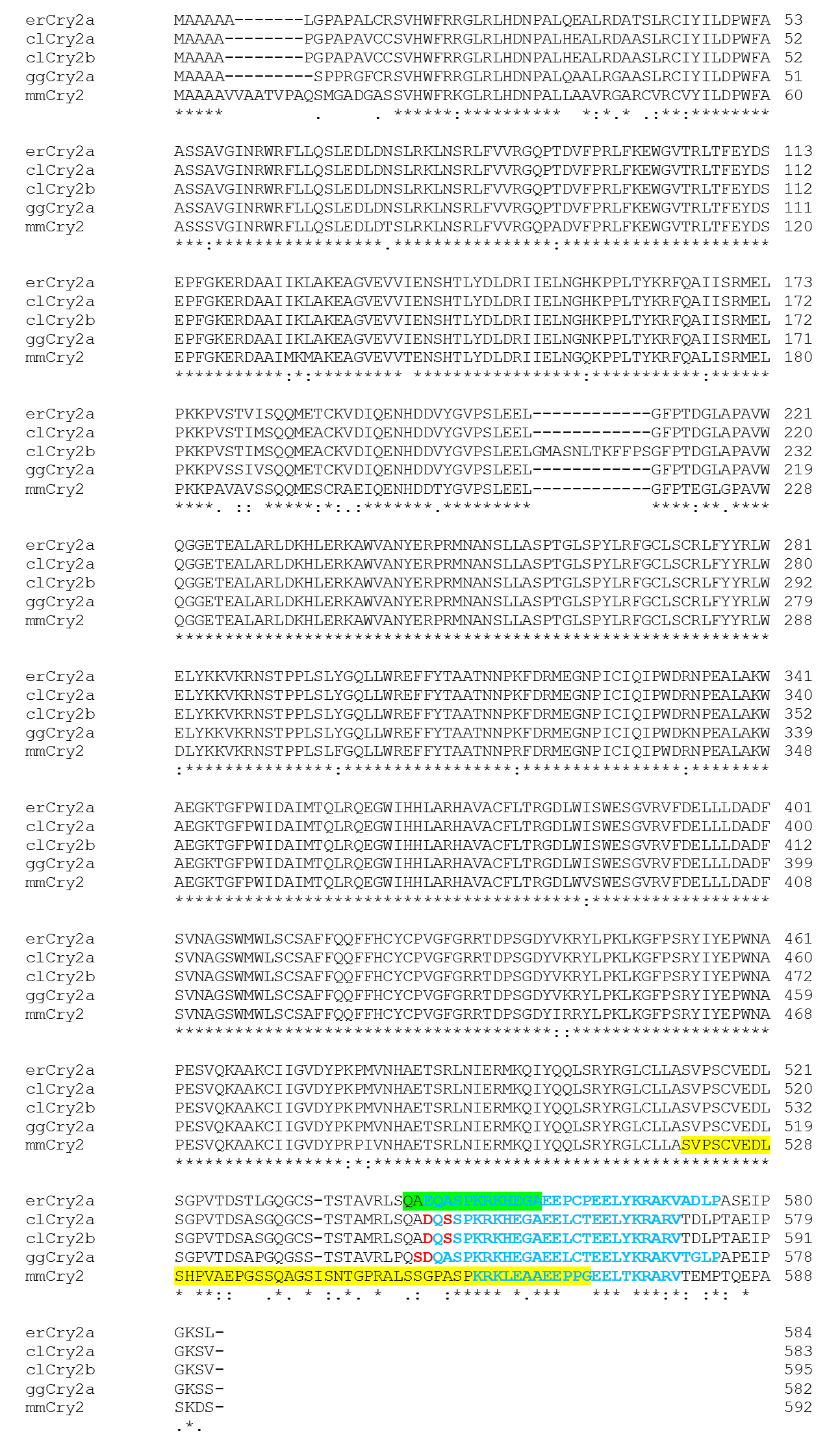

Supplement: Supplementary file 1 — Supplementary file1 (PNG 152 kb) [file 359_2021_1506_MOESM1_ESM.png]

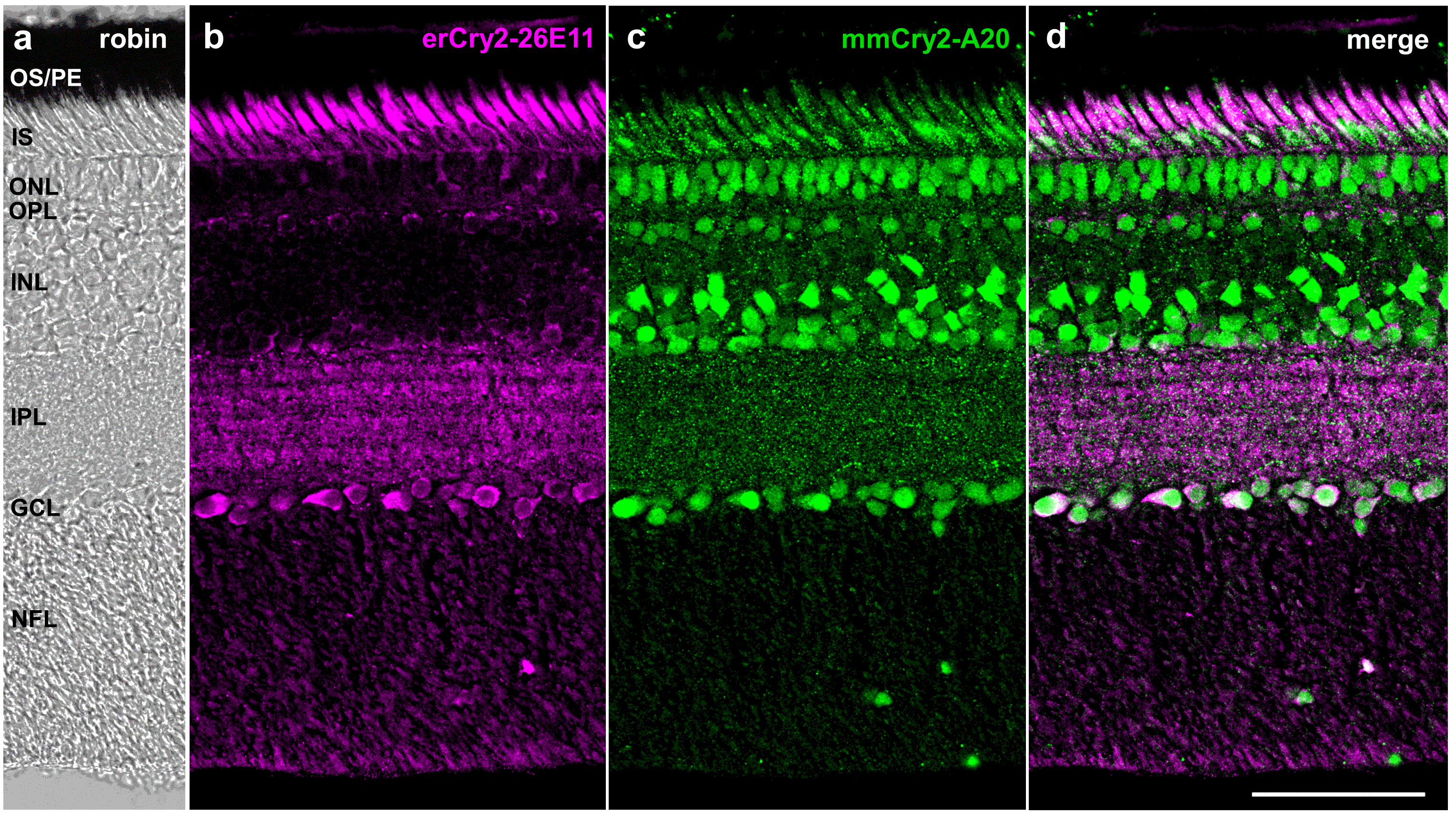

Supplement: Supplementary file 2 — Supplementary file2 (PNG 1281 kb) [file 359_2021_1506_MOESM2_ESM.png]

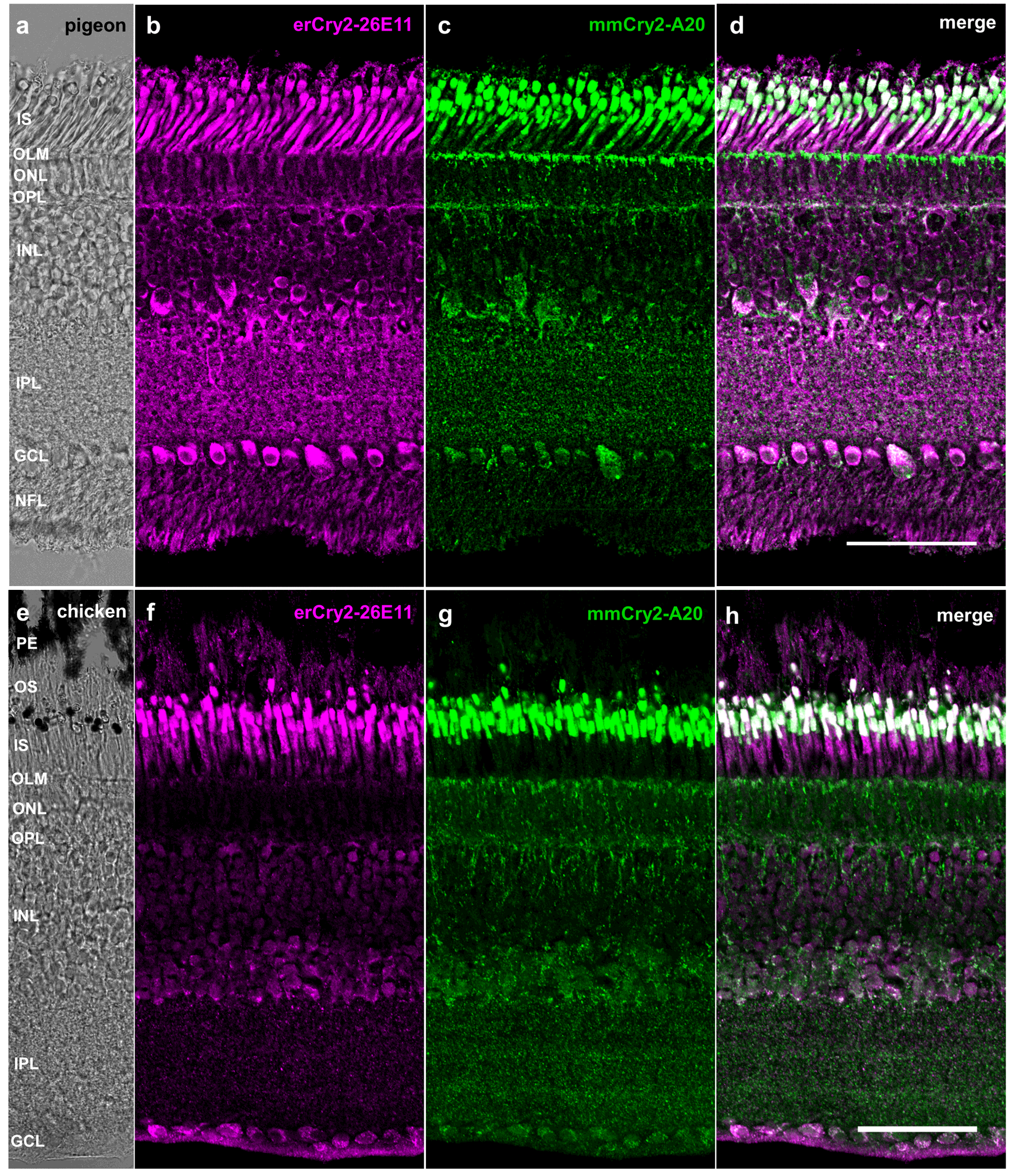

Supplement: Supplementary file 3 — Supplementary file3 (PNG 1571 kb) [file 359_2021_1506_MOESM3_ESM.png]

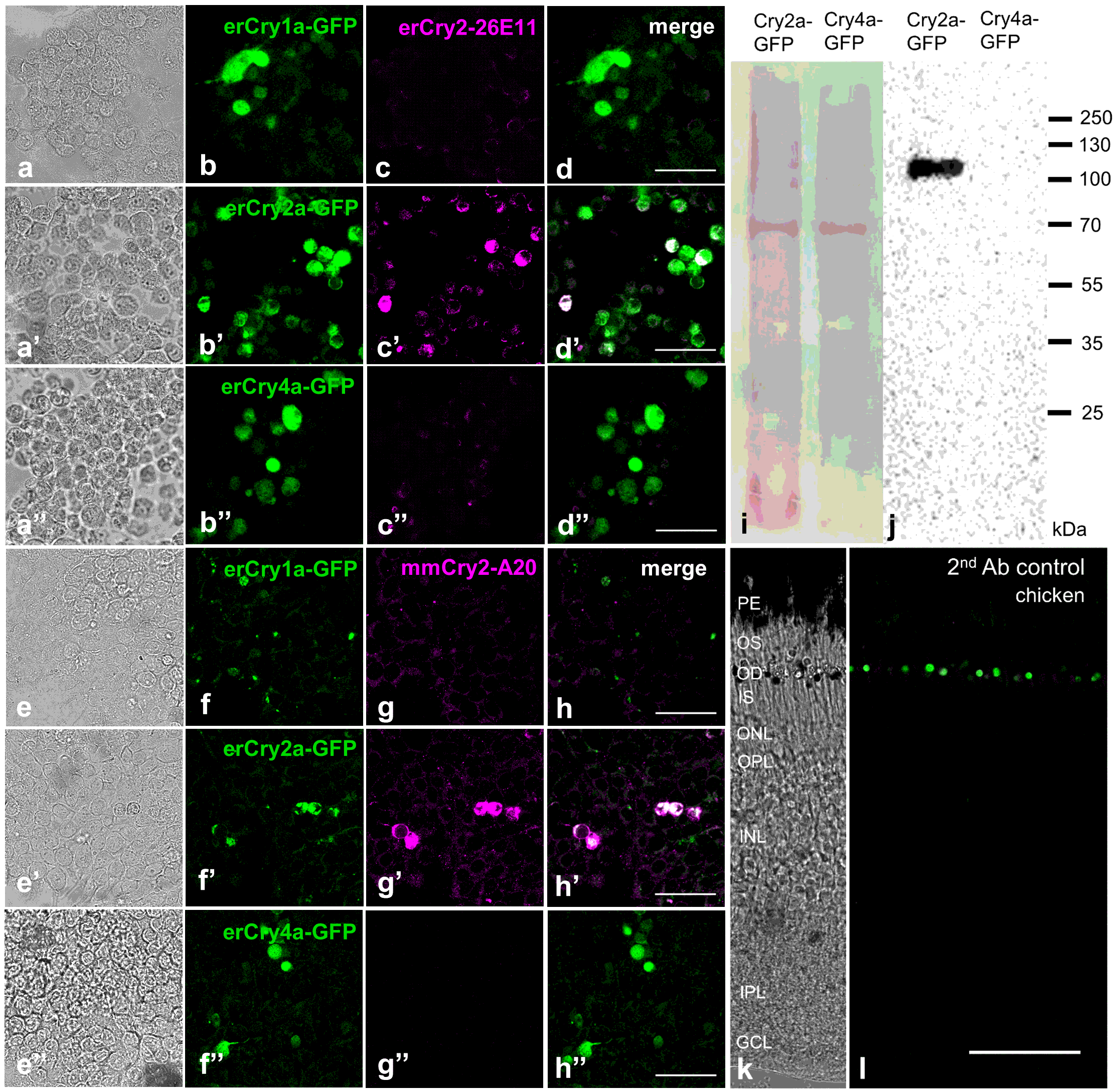

Supplement: Supplementary file 4 — Supplementary file4 (PNG 575 kb) [file 359_2021_1506_MOESM4_ESM.png]
