# Supplementary material for: Localisation of cryptochrome 2 in the avian retina
Source: J Comp Physiol A Neuroethol Sens Neural Behav Physiol. 2021 Oct 22;208(1):69–81. doi: 10.1007/s00359-021-01506-1 (PMC8918457; doi:10.1007/s00359-021-01506-1)
Supplement: Supplementary file 5 — Supplementary file5 (DOC 19553 kb) [file 359_2021_1506_MOESM5_ESM.doc]

**Supplementary information**

**Localisation of cryptochrome 2 in the avian retina**

*Journal of Comparative Physiology A*

Angelika Einwich^1^, Pranav Kumar Seth^1^, Rabea Bartölke^1^, Petra Bolte^1^, Regina Feederle^3^, Karin Dedek^1,2^, Henrik Mouritsen^1,2,*^

^1^Institute for Biology and Environmental Sciences, Carl von Ossietzky University Oldenburg, Oldenburg, Germany

^2^Research Centre for Neurosensory Sciences, Carl von Ossietzky University Oldenburg, Oldenburg, Germany

^3^Helmholtz Zentrum München, German Research Center for Environmental Health, Institute for Diabetes and Obesity, Monoclonal Antibody Core Facility, Neuherberg, Germany

***** Corresponding author

E-mail: henrik.mouritsen@uni-oldenburg.de (HM)

**
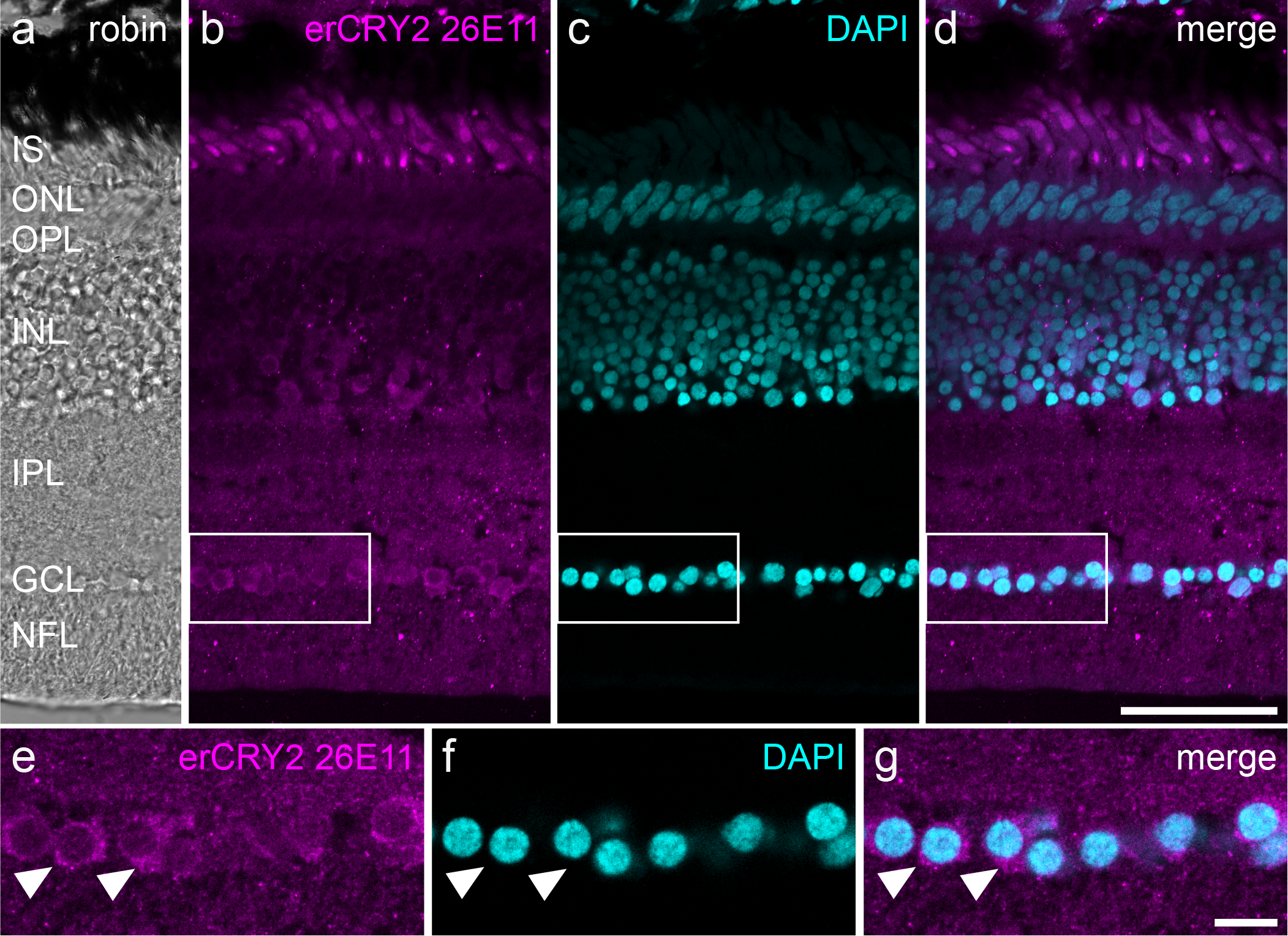
**

**Fig. S1 Subcellular localisation of Cry2 in the European robin retina**. Using the monoclonal erCry2-26E11 antibody together with the nuclear marker DAPI, Cry2 was detected in the cytoplasm of photoreceptor inner segments, inner nuclear layer and ganglion cell layer of the European robin retina (b, d). As expected, there was no Cry2 staining observed in the outer nuclear layer since the layer contains almost no cytoplasm (c). Since DAPI recognises the nuclear region of the cell layers and Cry2 seems to be predominantly located in the cytoplasm, b and c overlap only partially. Images e-g depict an enlarged image of the ganglion cell layer showing the partial overlap of Cry2 (e) and nuclear DAPI (f). However, cytoplasmic Cry2 is clearly visible (arrowheads). Image a is a bright field transmission image. Images B-G are maximum projections of confocal stacks. Please note that intensity was enhanced for magnified images (e-g) for better visibility. Scale bars: d, 50 µm; g, 10 µm. IS, photoreceptor inner segments; ONL, outer nuclear layer; OPL, outer plexiform layer; INL, inner nuclear layer; IPL, inner plexiform layer; GCL, ganglion cell layer; NFL, nerve fibre layer.

**
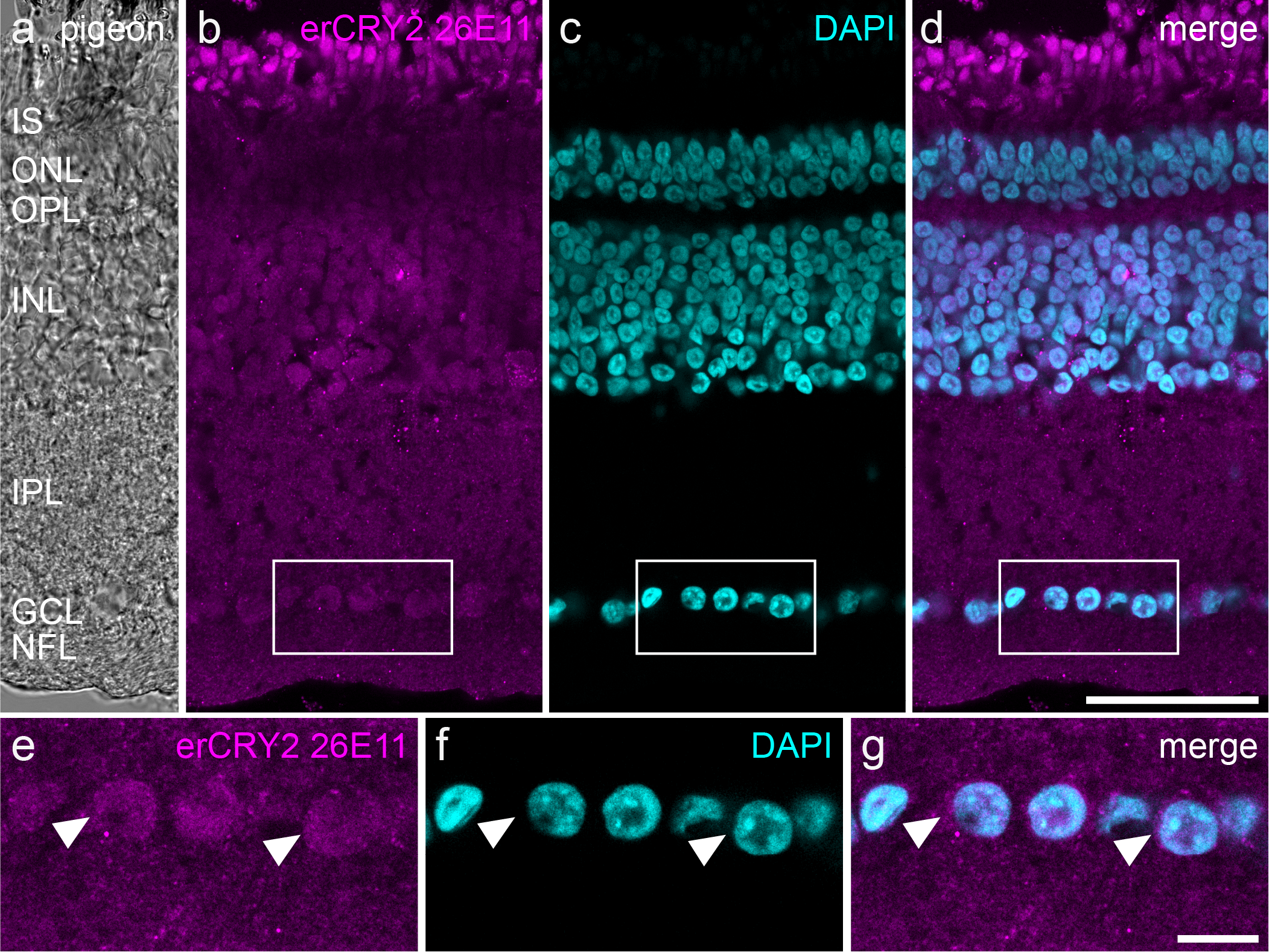
**

**Fig. S2 Subcellular localisation of Cry2 in the pigeon retina**. Using the monoclonal erCry2-26E11 antibody together with the nuclear marker DAPI, we observed Cry2 signal in the photoreceptor inner segments, inner nuclear layer and ganglion cell layer of the pigeon retina (b, d). Staining was most prominent in the cytoplasm, but Cry2 and DAPI also partially overlapped. However, labelling was lacking in the outer nuclear layer. Images e-g depict an enlarged image of the ganglion cell layer showing the partial overlap of Cry2 (e) and nuclear DAPI (f). Cytoplasmic Cry2 is clearly visible (arrowheads). Image a is a bright field transmission image. Images b-g are maximum projections of confocal stacks. Intensity was enhanced for magnified images (e-g) for better visibility. Scale bars: d, 50 µm; g, 10 µm. IS, photoreceptor inner segments; ONL, outer nuclear layer; OPL, outer plexiform layer; INL, inner nuclear layer; IPL, inner plexiform layer; GCL, ganglion cell layer; NFL, nerve fibre layer.


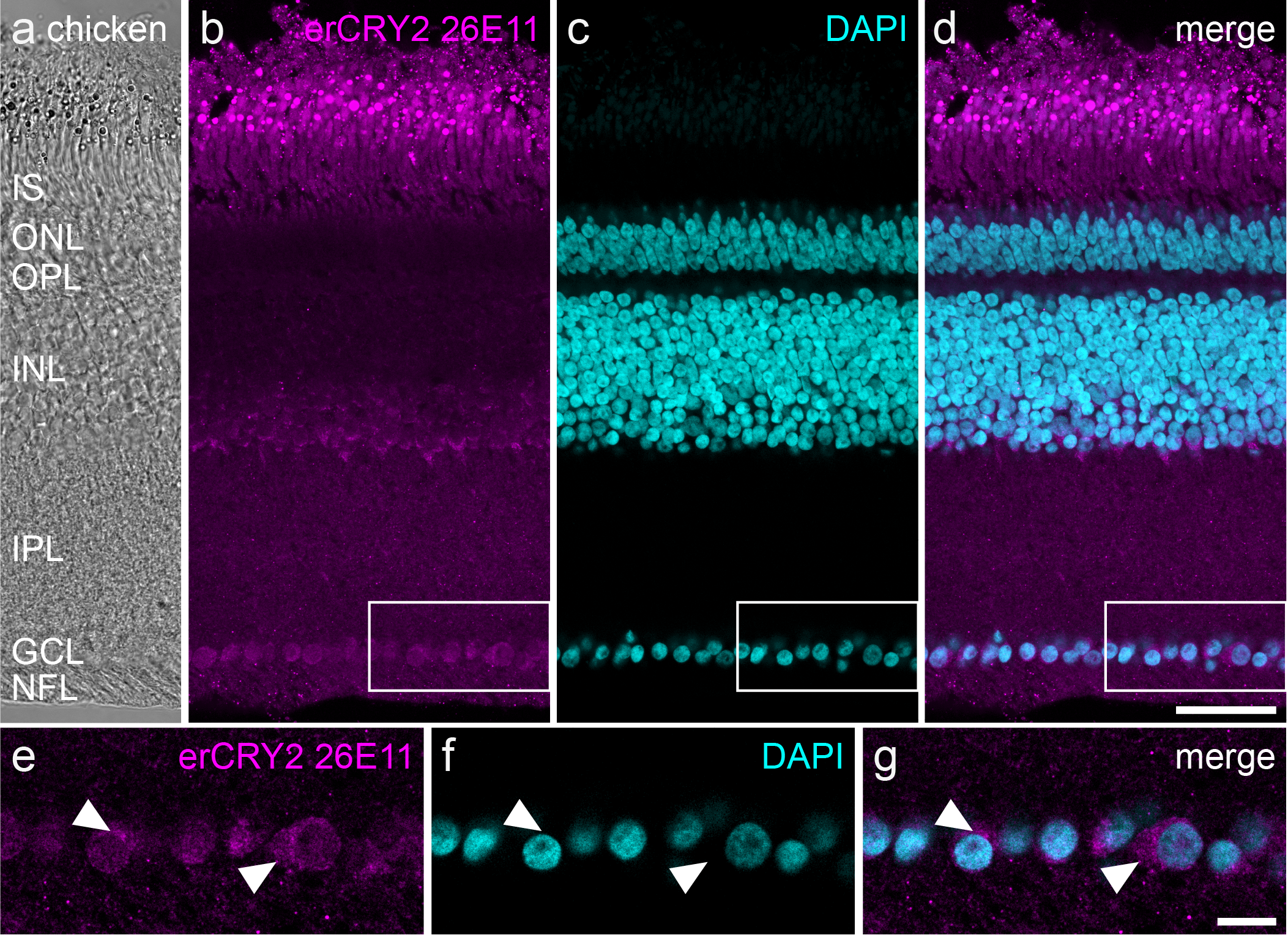


**Fig. S3 Subcellular localisation of Cry2 in the chicken retina**. Applying the monoclonal erCry2-26E11 antibody together with the nuclear marker DAPI on the chicken retina (a), we obtained Cry2 signal in the cytoplasm of photoreceptor inner segments, inner nuclear layer and ganglion cell layer (b, d). In the ganglion cell layer, weak nuclear Cry2 staining became visible. Images e-g depict an enlarged image of the ganglion cell layer showing the partial overlap of Cry2 (e) and nuclear DAPI (f). Cytoplasmic Cry2 (arrowheads) and nucleic Cry2 can be differentiated. Image a is a bright field transmission image. Images b-g are maximum projections of confocal stacks. Intensity was enhanced for magnified images (e-g) for better visibility. Scale bars: d, 50 µm; g, 10 µm. IS, photoreceptor inner segments; ONL, outer nuclear layer; OPL, outer plexiform layer; INL, inner nuclear layer; IPL, inner plexiform layer; GCL, ganglion cell layer; NFL, nerve fibre layer.


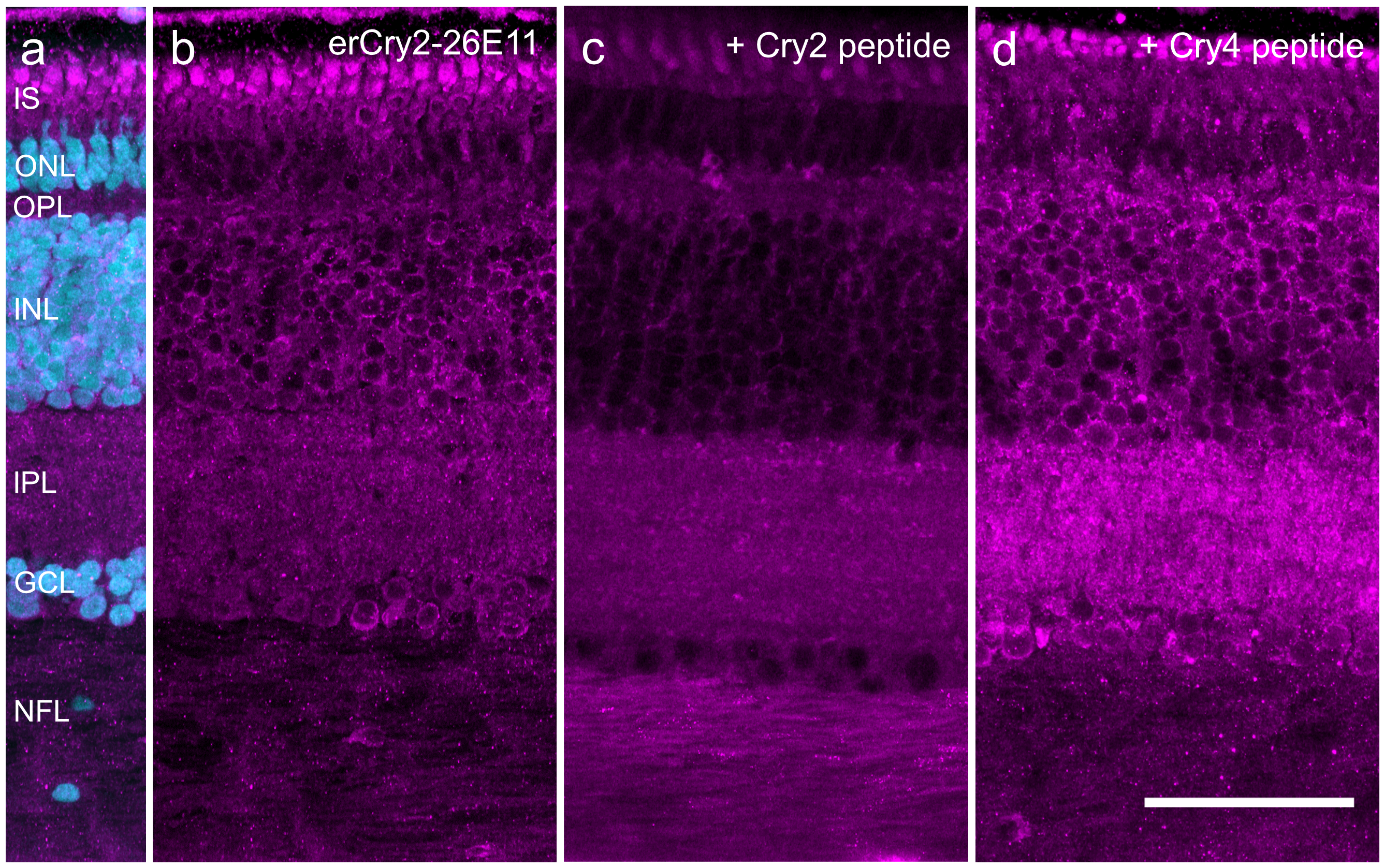


**Fig. S4 Pre-adsorption control on European robin retina.** (a, b) Labelling of the European robin retina with the monoclonal antibody erCry2-26E11 (magenta) shows antibody staining in the photoreceptor inner segments and the nuclear layers, co-stained with the nuclear maker DAPI (a). When pre-adsorbing the antibody with 500 µg/ml of the erCry2 peptide used for immunisation, the antibody shows no staining anymore (c), as compared with the original labelling (b). Only faint labelling of the inner segments is still visible (c). In contrast, Cry2 staining was unchanged after incubation with the same concentration of an erCry4 peptide (d). The images are maximum projections of confocal stacks. Scale bar 50 µm. IS, photoreceptor inner segments; ONL, outer nuclear layer; OPL, outer plexiform layer; INL, inner nuclear layer; IPL, inner plexiform layer; GCL, ganglion cell layer; NFL, nerve fibre layer.


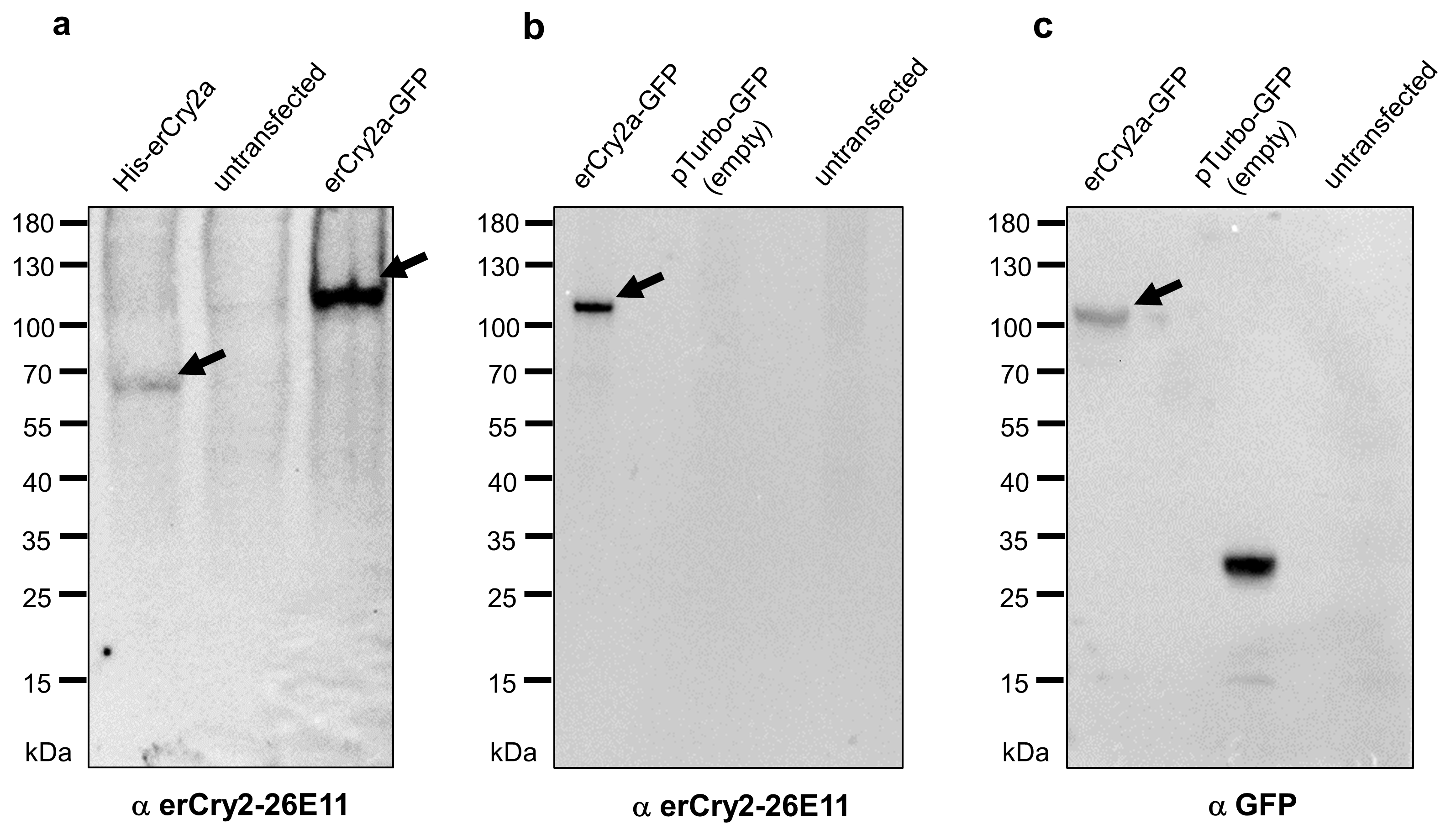


**Fig. S5 Immunoblots validating the specificity of the anti-erCry2-26E11 antibody.** To confirm the specificity of the erCry2-26E11 antibody, we produced both the erCry2a protein tagged with a His tag (a, left lane; His-erCry2a in the pcDNA3.1 vector) and fused to GFP (a, right lane; erCry2a-GFP in the pTurbo-GFP vector). Staining of HEK cell lysates overexpressing these two erCry2a fusion proteins with the erCry2-26E11 antibody demonstrates that the antibody recognizes the erCry2a protein irrespective of the tag (a, arrows). Parallel labelling of HEK cell lysates overexpressing only the erCry2a-GFP fusion protein with the erCry2-26E11 antibody (b) and with a GFP antibody (c) shows that the erCry2a-GFP band (b, arrow) is also recognised by the GFP antibody (c, arrow). The erCry2a-GFP fusion protein runs about 20 kDa higher than expected from its calculated size of 93.4 kDa (erCry2a 66.4 kDa, GFP 27 kDa). The empty pTurbo-GFP vector is only recognised by the anti-GFP antibody (c, middle lane), but not by the erCry2-26E11 antibody (b, middle lane). Untransfected HEK cell lysates served as negative controls in all blots. For panels a and b, the pellets of the protein samples, whereas for panel c, the supernatant portions were used for the immunoblot. Note that the His-erCry2a protein was expressed containing the entire *Cry2a* sequence whereas the erCry2a-GFP protein lacks the first 39 nucleotides.
